# CDTD-Agent: a retrieval-augmented multi-agent digital health framework for integrated diabetes care: a single-case expert-rated pilot study

**DOI:** 10.3389/fdgth.2026.1923975

**Published:** 2026-09-16

**Authors:** Jieyi Zhang, Luqing Wu, Jielong Wu, Xiaoming Han, Lijin Lin, Nengjiang Zhao, Zhihai Zhang, Miaona Cai, Meiqi Chen, Shuyu Yang, Bo Li

**Affiliations:** 1 Center of Integrated Chinese and Western Medicine, The First Affiliated Hospital of Xiamen University, Xiamen, Fujian, China; 2 Xiamen ITG Health Technology Co., Ltd., Xiamen, Fujian, China; 3 Department of Artificial Intelligence, School of Electrical Engineering and Automation, Xiamen University of Technology, Xiamen, Fujian, China

**Keywords:** diabetes management, large language models (LLMs), multi-agent system, retrieval-augmented generation (RAG), traditional Chinese medicine (TCM)

## Abstract

**Background:**

Diabetes management increasingly requires coordination across Western medicine, traditional Chinese medicine (TCM), and lifestyle-based health management. General-purpose large language models (LLMs) can generate clinical text but may lack reliable source grounding and structured cross-disciplinary reasoning.

**Methods:**

We developed CDTD-Agent, a retrieval-augmented multi-agent framework that combines a domain-specific knowledge base with an Extract-Retrieve-Reason workflow. The knowledge base included 12 medical textbooks, 8 clinical guidelines, and 183 expert-reviewed diabetes cases. In a blinded single-case pilot evaluation, 30 senior experts rated anonymized recommendations from CDTD-Agent, GPT-4o, DeepSeek-V3.2-Exp, Doubao, and two attending physicians across eight dimensions. Linear mixed-effects models accounted for repeated ratings within evaluators, with Holm correction across eight omnibus tests and global Benjamini-Hochberg correction across 40 focused CDTD-Agent-versus-comparator contrasts. A *post hoc* backbone-only ablation compared the full framework with Qwen3–235B-A22B using the same case and external prompt without retrieval or role-structured multi-agent processing.

**Results:**

Significant source effects were detected in all eight dimensions after Holm correction (all adjusted *p* < 0.001). After global false discovery rate correction, CDTD-Agent received significantly higher ratings than all three general-purpose LLMs in five dimensions: safety, examination appropriateness, Western medicine treatment appropriateness, TCM treatment appropriateness, and evidence adherence. The largest differences occurred in Western medicine treatment appropriateness, where CDTD-Agent scored 4.07 ± 0.94 versus 1.37–1.60 for the general-purpose LLMs (all adjusted *p* < 0.001). Non-parametric sensitivity analyses agreed with the mixed-effects results for 39 of 40 focused contrasts. In the *post hoc* ablation, the full framework outperformed the backbone-only condition in seven of eight dimensions after Holm correction; health management was not significantly different (adjusted *p* = 0.080).

**Conclusion:**

In this standardized complex T2DM case, CDTD-Agent received favorable expert ratings relative to the evaluated general-purpose LLMs in several clinically important dimensions. These findings are scenario-specific and do not establish generalizable clinical performance or isolate the effects of individual framework components. Multicase, repeated-generation, factorial-ablation, external, and prospective validation is needed before clinical deployment.

## 1 Introduction

Diabetes mellitus (DM) is a complex metabolic disorder and a major global public health challenge. The 11th edition of the International Diabetes Federation Diabetes Atlas estimated that 589 million adults aged 20–79 years were living with diabetes in 2024 and projected this number to increase to 853 million by 2050; China accounted for an estimated 148 million adults, the highest national total (1). Separately, Global Burden of Disease 2021 modeling projected that more than 1.31 billion people could be living with diabetes worldwide by 2050 (2). Diabetes reduces life expectancy and quality of life and imposes a substantial economic burden on individuals and health systems in China (3).

Effective diabetes management requires coordinated biomedical diagnosis, pharmacotherapy, and lifestyle-based health management (4), while integrated care involving TCM additionally requires individualized syndrome assessment and treatment planning (5). National and international guidance emphasizes comprehensive management that includes medical treatment, nutrition, exercise, and psychosocial support (4, 6), while recent TCM evidence reviews support consideration of complementary individualized approaches (5). Evidence from the co-management under three disciplines (CDTD) model suggests potential benefits for glycemic control and quality of life (7). However, implementing multidisciplinary care can remain difficult in resource-constrained primary-care settings (8). These implementation challenges create a need for accessible decision-support tools that can organize chronic-disease and integrative-care knowledge in a traceable manner (9, 10).

Artificial intelligence (AI) has been increasingly applied across diabetes care, including continuous glucose monitoring analysis, diabetic retinopathy screening, medical dialogue systems, and patient-facing health consultation (11–14). Large language models (LLMs) are particularly relevant because they can process free-text clinical information and generate structured explanatory responses. Digital TCM research has also increasingly explored structured and computational representations of TCM knowledge, including syndrome differentiation and herbal medicine (15).

Despite this potential, general-purpose LLMs have important limitations in high-stakes diabetes decision support. First, models without sufficient domain grounding may generate inaccurate or unsupported recommendations, particularly for complex treatment decisions and contraindication screening (16). Second, performance can vary substantially on highly specialized clinical tasks when knowledge or reasoning demands extend beyond general-purpose training (17). Third, integrated diabetes care requires reasoning across heterogeneous knowledge systems, including evidence-based Western medicine and TCM syndrome differentiation. Structured knowledge representation and domain-specific AI approaches may help organize such heterogeneous information and support more coherent clinical reasoning (18, 19).

To address these clinical and technical gaps, we developed CDTD-Agent, a retrieval-augmented multi-agent digital health framework for integrated diabetes care recommendations. The system combines a domain-specific knowledge base covering Western medical guidelines, TCM resources, and health-management protocols with an Extract-Retrieve-Reason workflow. A lightweight model first converts unstructured patient information into structured clinical entities, a hybrid retrieval module then selects relevant evidence, and a larger reasoning model generates recommendations through three role-defined agents representing endocrinology, TCM practice, and health management. We conducted a blinded, single-case pilot expert-rating evaluation against three general-purpose LLMs and two attending physicians using one standardized complex T2DM case. The objective was to examine the feasibility and expert-rated output quality of the complete framework under a defined clinical scenario, rather than to establish generalizable performance across the broader population of patients with diabetes.

## 2 Materials and methods

### 2.1 Data collection and knowledge base construction

To support cross-disciplinary reasoning, we constructed a structured domain-specific knowledge base covering Western medicine, traditional Chinese medicine (TCM), and health management. The knowledge base was derived from three main source categories. First, 12 core medical textbooks, including Internal Medicine, Diagnostics, TCM Internal Medicine, and Chinese Medical Formulae, were included to provide foundational knowledge on diabetes pathophysiology, diagnostic criteria, TCM syndrome differentiation, and treatment principles. Second, eight authoritative clinical guidelines and expert consensuses were incorporated to align the system with current evidence-based practice, including the Guideline for the Prevention and Treatment of Type 2 Diabetes in China (2024), the National Guide for Grassroots TCM Management of Diabetes (2022), and the ADA Standards of Care in Diabetes. Third, 183 diabetes and diabetes-complication cases were curated from the China Clinical Cases Library of TCM (CCCL-TCM). These expert case records from clinicians in different regions of China were manually selected to provide representative material for TCM syndrome differentiation, therapeutic principles, prescriptions, and follow-up information. In the CCCL-TCM records used for corpus construction, different visits from the same patient were not represented as separate case records. All patient-related information was de-identified before use. The composition of the knowledge base is summarized in Table 1.

**Table 1:** Composition and characteristics of the domain-specific knowledge base.

Data Category	Data Source/Origin	Key Content & Features	Volume/Sample Size
Medical Textbooks	Core curricula from People's Medical Publishing House & China Press of TCM	Foundational theory of internal medicine, TCM diagnostics, formulaology, and pathological mechanisms of diabetes.	12 Volumes
Clinical Guidelines	NHC, ADA, WFCMS, and CMA	Evidence-based diagnostic criteria, standardized treatment pathways, and expert consensus on complications.	8 Authoritative Guidelines
Standardized TCM Cases	CCCL-TCM	High-quality structured cases containing “Four Diagnostics,” syndrome differentiation, herbal prescriptions, and outcomes.	183 Expert-Reviewed Cases

NHC, national health commission; ADA, American Diabetes Association; WFCMS, World Federation of Chinese Medicine Societies; CMA, Chinese Medical Association; CCCL-TCM, China clinical cases library of TCM.

### 2.2 Data preprocessing and vector indexing

We established a retrieval subsystem that was independent of the generative model. Source documents were cleaned using rule-based preprocessing and converted into structured text units. Long documents were segmented into semantically coherent chunks using a sliding-window strategy, while metadata such as source type, title, section, and citation information were retained to support traceability. Each chunk was embedded using BGE-M3-Medical, a BERT-based embedding model adapted for Chinese medical text, and mapped into a 1024-dimensional dense vector space. The resulting embeddings were stored in a vector database for nearest-neighbor retrieval using cosine similarity. This design allowed the retrieval module to provide source-linked evidence fragments to the downstream reasoning model.

### 2.3 Retrieval-augmented generation architecture

To improve source grounding and support cross-disciplinary reasoning, we designed a three-stage Extract-Retrieve-Reason workflow using Qwen3–30B-A3B for structured clinical information extraction and Qwen3–235B-A22B as the large-model reasoning backbone.

First, in the clinical information extraction phase, Qwen3–30B-A3B processed unstructured patient descriptions, including symptoms, signs, laboratory indicators, and medical history. The model performed zero-shot information extraction and converted the input into structured clinical query objects. This step was intended to reduce irrelevant input noise and provide standardized retrieval queries for the subsequent retrieval module.

Second, in the hybrid retrieval phase, the structured query objects were submitted to a retrieval module that combined dense vector retrieval with sparse keyword matching. The system initially retrieved the top 20 candidate fragments from the knowledge base. A reranking model then scored the candidate fragments for semantic relevance, and the top five evidence fragments were retained as augmented context for generation. These fragments could include Western guideline recommendations, TCM knowledge, or similar expert-reviewed cases.

Third, in the multi-role reasoning and generation phase, the retrieved evidence fragments were inserted into the prompt context and Qwen3–235B-A22B was used as the reasoning model. The model generated responses through three role-defined agents: an endocrinologist, a TCM practitioner, and a health manager. The system used in-context learning rather than parameter fine-tuning. To improve traceability, the prompt required key recommendations, such as pharmacotherapy suggestions and recommended examinations, to cite the retrieved evidence used to support them.

### 2.4 Prompt engineering

We developed a prompt framework based on four elements: role, task, context, and output format. The role component assigned the model a discipline-specific perspective, such as endocrinologist, TCM practitioner, or health manager. The task component specified the required output, including diagnostic assessment, Western medicine treatment recommendations, TCM syndrome differentiation and treatment, and lifestyle-management recommendations. The context component provided the retrieved evidence fragments and structured clinical entities. The output-format component required the model to generate a structured care recommendation with explicit evidence references where applicable.

Separate instruction templates were used for the three disciplinary roles. The Western medicine template emphasized evidence-based diagnosis, risk assessment, contraindication screening, and pharmacotherapy recommendations grounded in retrieved guidelines. The TCM template followed the reasoning sequence of principle, method, prescription, and medicinals (Li-Fa-Fang-Yao), based on the four diagnostic methods and syndrome differentiation. The health-management template focused on individualized diet, exercise, and behavioral recommendations. Safety constraints were included in each template, requiring the model to avoid unsupported remedies, identify potential contraindications, and state that the output was intended as decision support rather than a substitute for clinician judgment.

### 2.5 Workflow design

Figure 1 summarizes the overall CDTD-Agent architecture and evaluation framework, while Figure 2 provides a more detailed view of the end-to-end workflow from clinical input through evidence retrieval and multi-role synthesis.

**Figure 1:**
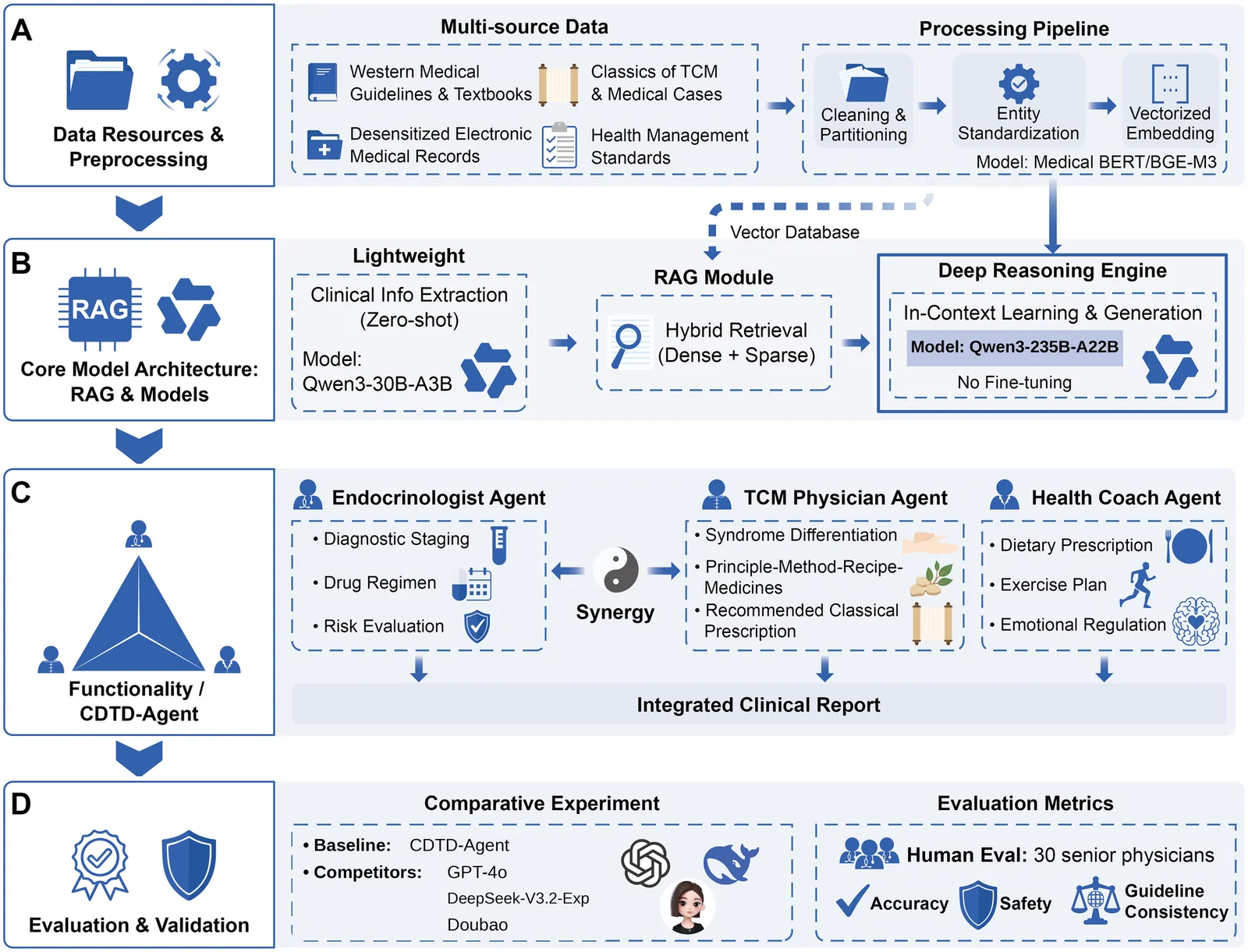
Architectural workflow of CDTD-Agent. The figure summarizes four components: **(A)** data-resource preparation and preprocessing; **(B)** structured clinical-information extraction, hybrid dense-and-sparse retrieval, and in-context generation; **(C)** role-structured reasoning by endocrinology, traditional Chinese medicine, and health-management agents followed by synthesis of an integrated clinical report; and **(D)** comparative expert evaluation and validation.

**Figure 2:**
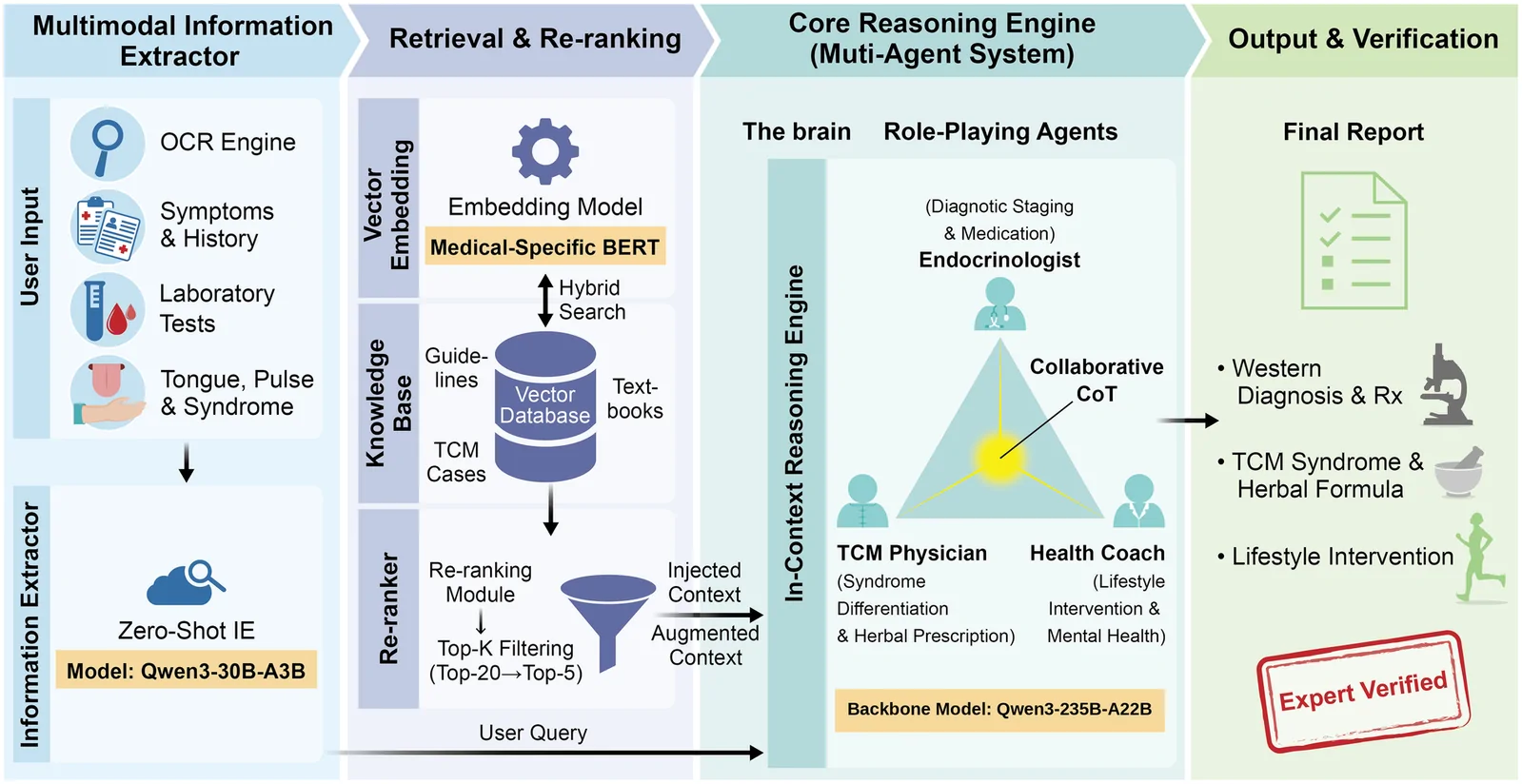
Four-stage operational workflow of CDTD-Agent in the expert-rating evaluation. The system extracts structured clinical information from patient input, retrieves and re-ranks relevant evidence, performs multi-role reasoning and synthesis, and produces an expert-verified integrated recommendation.

### 2.6 Evaluation methodology

#### 2.6.1 Single-case experimental design and sample selection

We conducted a blinded, controlled, single-case pilot expert-rating evaluation with evaluators recruited from multiple institutions to assess the quality of recommendations generated under a defined clinical scenario. One complex type 2 diabetes mellitus (T2DM) case from CCCL-TCM was used as the standardized test case. The case included history of present illness, past medical history, physical examination findings, and key laboratory indicators, including fasting plasma glucose, HbA1c, and islet function tests. It also involved complication-management requirements and therefore provided a clinically complex scenario requiring integrated Western medicine, TCM, and health-management reasoning. The case was used as a challenge scenario for this pilot evaluation and was not intended to represent the broader spectrum of T2DM phenotypes or clinical settings. A retrospective case-overlap assessment confirmed that the standardized test case was not included in the 183-case retrieval corpus and that no separately entered follow-up record of the same patient was present. To ensure consistent input across all comparison groups, the same case information was formatted into a standardized prompt template.

#### 2.6.2 Control models and generation process

The primary evaluation compared CDTD-Agent with three general-purpose LLMs—GPT-4o (20), DeepSeek-V3.2-Exp in non-thinking mode (21), and Doubao (22)—and two attending physicians, one from a tertiary hospital and one from a primary healthcare institution. Evaluation testing commenced on October 11, 2025. GPT-4o was explicitly selected in the official ChatGPT web interface; DeepSeek-V3.2-Exp was accessed through the official DeepSeek web interface with the “Deep Thinking” option disabled; and Doubao was accessed through its official consumer web interface. For Doubao, the specific backend model snapshot was not exposed by the consumer interface; the comparator is therefore reported at the product level. CDTD-Agent used Qwen3–235B-A22B as its large-model reasoning backbone and was locally deployed on computing infrastructure provided by Xiamen ITG Health Technology Co., Ltd., which participated in technical development under a commissioned research and development agreement for the project.

All AI systems received the identical standardized case information and the same external evaluation prompt, which required coverage of Western medicine care, TCM care, health management, patient-specific rationale for important decisions, and relevant contraindications or potential risks; the full prompt is reproduced in Supplementary Appendix S1. The two physicians received the same standardized case and were asked to prepare a comprehensive care plan covering the same clinical domains. No model-specific instructions were added to the external evaluation prompt for the general-purpose LLMs. After receiving the common case and task prompt, CDTD-Agent processed the input through its predefined Extract-Retrieve-Reason workflow, whereas the general-purpose LLMs generated responses using their native model capabilities.

A single complete output was generated from each AI system, and one recommendation from each physician was retained for expert evaluation. No repeated generations, best-of-n selection, or manual selection among multiple candidate AI outputs was performed. For the three web-based comparator systems, low-level decoding parameters such as temperature, top-p, maximum output tokens, and random seed were not user-configurable or exposed through the consumer interfaces. For the locally deployed CDTD-Agent, the exact decoding configuration used during the original generation was not retained. All outputs were anonymized before evaluation by removing model names, physician identifiers, and generation timestamps and randomly recoding the outputs before expert scoring.

#### 2.6.3 *Post hoc* backbone-only ablation analysis

To assess the incremental performance associated with the complete CDTD-Agent framework relative to the backbone model alone, we conducted a *post hoc* backbone-only ablation analysis using Qwen3–235B-A22B. The backbone-only condition received the same standardized case and external evaluation prompt as the full CDTD-Agent but did not use the retrieval corpus, retrieved evidence context, role-structured agent workflow, or multi-role synthesis process. The resulting fixed output was evaluated by the same 30 experts using the same eight 5-point Likert dimensions, with evaluator identifiers retained for pairing with the original CDTD-Agent ratings.

#### 2.6.4 Evaluation metrics and expert scoring

An independent evaluation committee comprising 30 clinical experts was established for this study. All members held senior professional titles (Associate Chief Physician or higher) and had extensive clinical experience in endocrinology or TCM internal medicine. The experts independently scored the anonymized recommendations using a standardized evaluation framework consisting of eight dimensions. Each dimension was rated on a 5-point Likert scale, ranging from 1 (completely inaccurate or unreasonable) to 5 (completely accurate or reasonable). The dimensions assessed diagnostic consistency, examination appropriateness, Western medicine treatment appropriateness, TCM treatment appropriateness, health-management feasibility, safety, evidence adherence, and personalization; definitions are provided in Table 2. Evidence Adherence represented the evaluators’ overall judgment of whether a recommendation followed evidence-based guideline principles and appeared to be supported by appropriate clinical evidence; it was not designed as formal verification of individual citations or retrieved evidence fragments. Data were collected using the Wenjuanxing online survey platform, and evaluators remained blinded to recommendation source throughout scoring.

**Table 2:** Definitions of evaluation dimensions for clinical treatment plans.

Dimension	Definition
Diagnostic Consistency	Alignment between the generated diagnosis and established clinical knowledge or guidelines.
Examination Appropriateness	Appropriateness and necessity of recommended examinations for disease assessment.
Western Medicine Treatment Appropriateness	Appropriateness of pharmacotherapy and biomedical treatment recommendations, including indications and contraindications.
TCM Treatment Appropriateness	Appropriateness of syndrome differentiation, therapeutic principles, prescriptions, and medicinals.
Health Management Feasibility	Scientific validity and practical feasibility of diet, exercise, and lifestyle recommendations.
Safety	Consideration of drug interactions, adverse reactions, contraindications, and potential clinical risks.
Evidence Adherence	Expert-rated overall alignment of the recommendation with evidence-based guidelines and whether major recommendations appeared to be supported by appropriate clinical evidence; this rating did not constitute citation-level verification.
Personalization	Extent to which recommendations reflect patient-specific age, complications, symptoms, and constitutional characteristics.

#### 2.6.5 *Post hoc* structured qualitative review of western medicine treatment outputs

A *post hoc* structured qualitative review was conducted on the original fixed outputs of the three general-purpose LLMs for Western medicine treatment. The three outputs were re-presented in anonymized form to two clinician reviewers, who independently assessed eight management domains defined for this review: (1) glycemic treatment intensity; (2) thyroid abnormality management; (3) hypertriglyceridemia/dyslipidemia management; (4) fatty liver and liver-function management; (5) polycystic ovary syndrome (PCOS) management; (6) diabetes complication screening; (7) hyperuricemia management; and (8) medication safety and follow-up monitoring. Each domain was coded as adequately addressed (A), partially addressed (P), not addressed (N), or potentially questionable recommendation (Q); a combined P/Q code was permitted when both partial coverage and a potentially questionable recommendation were present. The reviewers completed the assessments independently and subsequently discussed discordant items to reach consensus. The original independent assessments were retained in the study records, and the final consensus coding is reported in Supplementary Table S1. No inferential statistical testing was applied to this qualitative review.

#### 2.6.6 Ethical approval

This study was reviewed and approved by the Institutional Review Board of The First Affiliated Hospital of Xiamen University (Approval No. 2023–088). The requirement for written informed consent was waived for the use of anonymized case materials and expert evaluation data. No identifiable patient information was included in the study materials reported in this manuscript. All procedures were conducted in accordance with the Declaration of Helsinki and relevant Chinese guidelines for biomedical research.

#### 2.6.7 Statistical analysis

Because the same 30 evaluators independently rated all six recommendation sources across each of the eight evaluation dimensions, the resulting data had a repeated-measures structure, with ratings clustered within evaluators. The original study design did not prospectively specify a single primary outcome; therefore, all eight dimensions were treated as co-equal exploratory evaluation outcomes. Descriptive results are presented as mean ± standard deviation (SD). For the primary inferential analysis, a separate linear mixed-effects model was fitted for each evaluation dimension, with recommendation source entered as a fixed effect and evaluator entered as a random intercept. The overall source effect within each dimension was assessed using a joint Wald test with five degrees of freedom. To account for multiplicity across the eight exploratory dimensions, the eight omnibus *p* values were adjusted using the Holm procedure. Focused model-based contrasts compared CDTD-Agent with each of the five comparators within each dimension, yielding 40 contrasts in total; these *p*-values were jointly adjusted using the Benjamini-Hochberg false discovery rate procedure. Effect estimates are reported as mean differences (CDTD-Agent minus comparator) with 95% confidence intervals (CIs). Because the outcomes were measured on a 5-point ordinal Likert scale, Friedman tests followed by paired Wilcoxon signed-rank comparisons were additionally performed as non-parametric sensitivity analyses using the same multiplicity framework. Inter-rater reliability was summarized using two-way absolute-agreement intraclass correlation coefficients with 95% CIs, reporting both single-measure ICC [ICC(A,1)] and the reliability of the mean rating aggregated across all 30 evaluators [ICC(A,30)]. All tests were two-sided, and adjusted *p* < 0.05 was considered statistically significant. Statistical analyses were performed in R (version 4.6.1) and independently cross-checked in Python 3.11.

For the *post hoc* backbone-only ablation, a separate linear mixed-effects model was fitted for each dimension with system condition (full CDTD-Agent versus Qwen3–235B-A22B backbone-only) as a fixed effect and evaluator as a random intercept. The eight ablation *p*-values were adjusted using the Holm procedure, and effect estimates are reported as mean differences (CDTD-Agent minus Qwen backbone) with 95% CIs. Paired Wilcoxon signed-rank tests with Holm correction across the eight dimensions were used as a non-parametric sensitivity analysis.

## 3 Results

The primary evaluation compared recommendations generated for the same standardized diabetes case by CDTD-Agent, three general-purpose LLMs, and two attending physicians across eight dimensions. A *post hoc* backbone-only ablation then compared the full CDTD-Agent output with direct Qwen3–235B-A22B generation under the same case and external prompt. A separate *post hoc* structured qualitative review examined the content characteristics of the three general-purpose LLM outputs associated with low Western Medicine Treatment Appropriateness ratings.

### 3.1 Characteristics of expert evaluators

Thirty expert evaluators participated in the blinded scoring process. Of these, 22 were female (73.33%) and 8 were male (26.67%). Most evaluators were aged 31–50 years (25/30, 83.33%), and 25 (83.33%) held doctoral degrees. All were senior clinicians, including 18 Associate Chief Physicians (60.00%) and 12 Chief Physicians (40.00%). Most had more than 10 years of clinical experience, including 17 with 11–20 years of experience (56.67%) and 11 with more than 20 years of experience (36.67%). Twenty-eight evaluators (93.33%) were affiliated with Grade III Level A hospitals. Evaluator characteristics are summarized in Table 3. Single-evaluator absolute agreement varied substantially across the eight dimensions, with ICC(A,1) estimates ranging from 0.045 to 0.771. The highest single-evaluator agreement was observed for Western Medicine Treatment Appropriateness [ICC(A,1) = 0.771, 95% CI 0.556–0.953], whereas Diagnostic Consistency showed the lowest agreement [ICC(A,1) = 0.045, 95% CI 0.009–0.259]. Reliability was substantially higher when ratings were aggregated across the full 30-evaluator panel, with ICC(A,30) ranging from 0.585 to 0.990. Dimension-specific ICC estimates and 95% CIs are provided in Supplementary Table S2.

**Table 3:** Demographic and professional characteristics of expert evaluators.

Category	Subcategory	Count	Percentage (%)
Gender	Male	8	26.67%
	Female	22	73.33%
Age Group	≤24 yrs	0	0.00%
	25–30 yrs	0	0.00%
	31–40 yrs	10	33.33%
	41–50 yrs	15	50.00%
	51–60 yrs	5	16.67%
	≥61 yrs	0	0.00%
Educational Background	Bachelor's Degree	0	0.00%
	Master's Degree	5	16.67%
	Doctoral Degree	25	83.33%
Academic Title	Resident	0	0.00%
	Attending Physician	0	0.00%
	Associate Chief Physician	18	60.00%
	Chief Physician	12	40.00%
Clinical experience	≤5 yrs	1	3.33%
	6–10 yrs	1	3.33%
	11–20 yrs	17	56.67%
	≥21 yrs	11	36.67%
Hospital Grade	Grade III Level A hospital	28	93.33%
	Tertiary hospital	1	3.33%
	Secondary hospital	1	3.33%
	First level hospital	0	0.00%

### 3.2 Overall comparison of expert-rating scores

Table 4 and Figure 3 summarize descriptive expert ratings across the eight exploratory evaluation dimensions, while Table 5 and Figure 4 present the 40 focused model-based contrasts comparing CDTD-Agent with each of the five comparators. After Holm correction across the eight dimension-level omnibus tests, significant source effects were detected in all eight dimensions (all adjusted *p* < 0.001). After global Benjamini-Hochberg correction across the 40 focused contrasts, CDTD-Agent received significantly higher expert ratings than all three general-purpose LLMs in five dimensions: Safety, Examination Appropriateness, Western Medicine Treatment Appropriateness, TCM Treatment Appropriateness, and Evidence Adherence.

**Figure 3:**
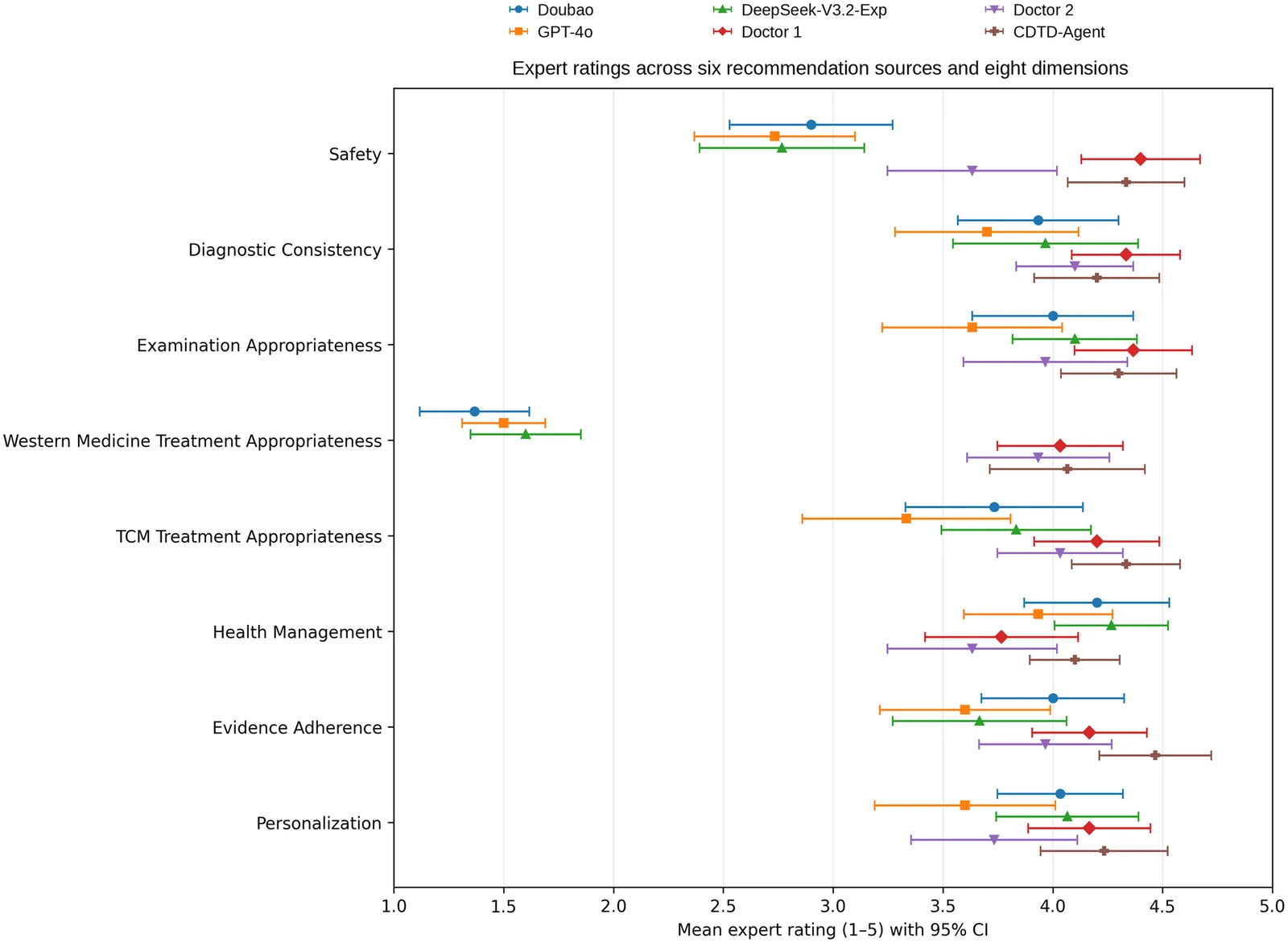
Expert ratings across six recommendation sources and eight evaluation dimensions. Points show mean ratings and 95% confidence intervals based on 30 evaluators. These descriptive intervals complement the mixed-effects inferential analyses reported in Tables 4, 5.

**Figure 4:**
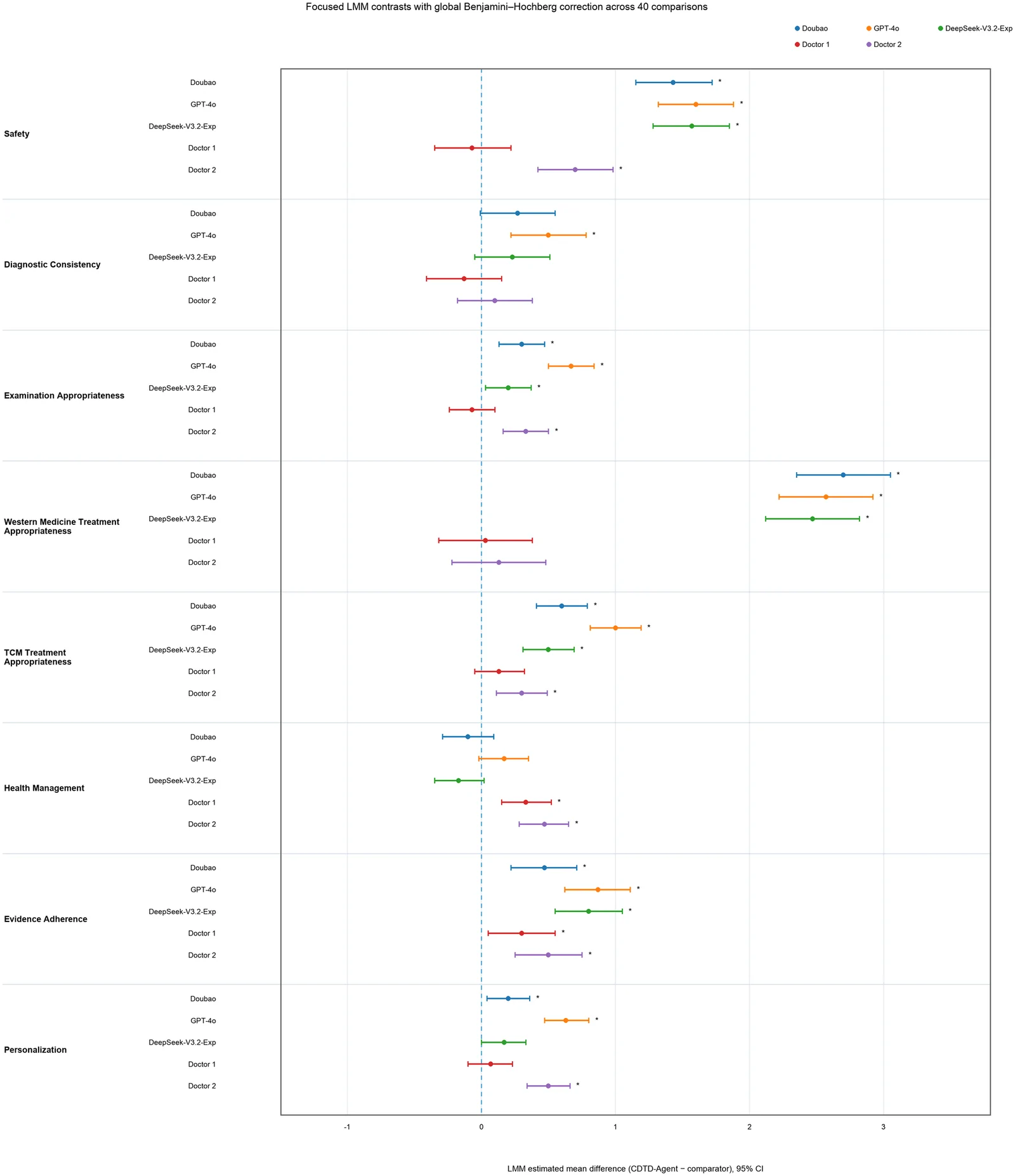
Focused linear mixed-effects model contrasts comparing CDTD-Agent with each comparator across eight dimensions. Values are estimated mean differences (CDTD-Agent minus comparator) with 95% confidence intervals. Asterisks indicate global Benjamini-Hochberg-adjusted *p* < 0.05 across the 40 focused contrasts. Positive values favor CDTD-Agent.

**Table 4:** Expert ratings and omnibus mixed-effects comparisons across eight exploratory evaluation dimensions.

Dimension	Doubao	GPT-4o	DeepSeek-V3.2-Exp	Doctor 1	Doctor 2	CDTD-Agent	Wald *χ*^2^	Holm-adjusted p
Safety	2.90 ± 0.99	2.73 ± 0.98	2.77 ± 1.01	4.40 ± 0.72	3.63 ± 1.03	4.33 ± 0.71	288.56	<0.001
Diagnostic Consistency	3.93 ± 0.98	3.70 ± 1.12	3.97 ± 1.13	4.33 ± 0.66	4.10 ± 0.71	4.20 ± 0.76	24.07	<0.001
Examination Appropriateness	4.00 ± 0.98	3.63 ± 1.10	4.10 ± 0.76	4.37 ± 0.72	3.97 ± 1.00	4.30 ± 0.70	92.16	<0.001
Western Medicine Treatment Appropriateness	1.37 ± 0.67	1.50 ± 0.51	1.60 ± 0.67	4.03 ± 0.76	3.93 ± 0.87	4.07 ± 0.94	597.14	<0.001
TCM Treatment Appropriateness	3.73 ± 1.08	3.33 ± 1.27	3.83 ± 0.91	4.20 ± 0.76	4.03 ± 0.76	4.33 ± 0.66	141.76	<0.001
Health Management	4.20 ± 0.89	3.93 ± 0.91	4.27 ± 0.69	3.77 ± 0.94	3.63 ± 1.03	4.10 ± 0.55	69.95	<0.001
Evidence Adherence	4.00 ± 0.87	3.60 ± 1.04	3.67 ± 1.06	4.17 ± 0.70	3.97 ± 0.81	4.47 ± 0.68	65.44	<0.001
Personalization	4.03 ± 0.76	3.60 ± 1.10	4.07 ± 0.87	4.17 ± 0.75	3.73 ± 1.01	4.23 ± 0.77	88.73	<0.001

Values are presented as mean ± standard deviation (SD) based on ratings from 30 evaluators. For each evaluation dimension, a linear mixed-effects model was fitted with recommendation source as a fixed effect and evaluator as a random intercept. The omnibus source effect was assessed using a joint Wald test with 5 degrees of freedom. Because no single primary outcome was prospectively specified, all eight dimensions were treated as exploratory outcomes, and the eight omnibus *p*-values were adjusted using the Holm procedure. LLM, large language model; TCM, traditional Chinese medicine.

**Table 5:** Focused mixed-effects comparisons between CDTD-Agent and each comparator.

Dimension	vs. Doubao	vs. GPT-4o	vs. DeepSeek-V3.2-Exp	vs. Doctor 1	vs. Doctor 2
Safety	1.43 [1.15, 1.72]; *p* < 0.001	1.60 [1.32, 1.88]; *p* < 0.001	1.57 [1.28, 1.85]; *p* < 0.001	−0.07 [−0.35, 0.22]; *p* = 0.660	0.70 [0.42, 0.98]; *p* < 0.001
Diagnostic Consistency	0.27 [-0.01, 0.55]; *p* = 0.090	0.50 [0.22, 0.78]; *p* < 0.001	0.23 [-0.05, 0.51]; *p* = 0.134	−0.13 [-0.41, 0.15]; *p* = 0.415	0.10 [-0.18, 0.38]; *p* = 0.511
Examination Appropriateness	0.30 [0.13, 0.47]; *p* = 0.001	0.67 [0.50, 0.84]; *p* < 0.001	0.20 [0.03, 0.37]; *p* = 0.033	−0.07 [−0.24, 0.10]; *p* = 0.492	0.33 [0.16, 0.50]; *p* < 0.001
Western Medicine Treatment Appropriateness	2.70 [2.35, 3.05]; *p* < 0.001	2.57 [2.22, 2.92]; *p* < 0.001	2.47 [2.12, 2.82]; *p* < 0.001	0.03 [−0.32, 0.38]; *p* = 0.852	0.13 [−0.22, 0.48]; *p* = 0.494
TCM Treatment Appropriateness	0.60 [0.41, 0.79]; *p* < 0.001	1.00 [0.81, 1.19]; *p* < 0.001	0.50 [0.31, 0.69]; *p* < 0.001	0.13 [−0.05, 0.32]; *p* = 0.204	0.30 [0.11, 0.49]; *p* = 0.003
Health Management	−0.10 [-0.29, 0.09]; *p* = 0.352	0.17 [−0.02, 0.35]; *p* = 0.104	−0.17 [-0.35, 0.02]; *p* = 0.104	0.33 [0.15, 0.52]; *p* < 0.001	0.47 [0.28, 0.65]; *p* < 0.001
Evidence Adherence	0.47 [0.22, 0.71]; *p* < 0.001	0.87 [0.62, 1.11]; *p* < 0.001	0.80 [0.55, 1.05]; *p* < 0.001	0.30 [0.05, 0.55]; *p* = 0.028	0.50 [0.25, 0.75]; *p* < 0.001
Personalization	0.20 [0.04, 0.36]; *p* = 0.028	0.63 [0.47, 0.80]; *p* < 0.001	0.17 [0.00, 0.33]; *p* = 0.071	0.07 [−0.10, 0.23]; *p* = 0.489	0.50 [0.34, 0.66]; *p* < 0.001

Estimated mean differences are expressed as CDTD-Agent minus comparator; positive values favor CDTD-Agent. Values are reported as estimated mean differences with 95% confidence intervals (CIs). The 40 CDTD-Agent-versus-comparator contrasts (8 dimensions  ×  5 comparators) were jointly adjusted using the Benjamini-Hochberg false discovery rate procedure. All tests were two-sided; global BH-adjusted *p* < 0.05 was considered statistically significant.

#### 3.2.1 Safety

CDTD-Agent scored 4.33 ± 0.71. It received significantly higher ratings than Doubao (mean difference 1.43, 95% CI 1.15–1.72), GPT-4o (1.60, 95% CI 1.32–1.88), and DeepSeek-V3.2-Exp (1.57, 95% CI 1.28–1.85; all global BH-adjusted *p* < 0.001). CDTD-Agent also scored higher than Doctor 2 (0.70, 95% CI 0.42–0.98; adjusted *p* < 0.001), whereas no significant difference was detected relative to Doctor 1 (−0.07, 95% CI −0.35 to 0.22; adjusted *p* = 0.660).

#### 3.2.2 Diagnostic consistency

CDTD-Agent scored 4.20 ± 0.76. Among the three general-purpose LLMs, a statistically significant difference was observed only versus GPT-4o (mean difference 0.50, 95% CI 0.22–0.78; adjusted *p* < 0.001). Differences versus Doubao (0.27, 95% CI -0.01 to 0.55; adjusted *p* = 0.090) and DeepSeek-V3.2-Exp (0.23, 95% CI −0.05 to 0.51; adjusted *p* = 0.134) were not statistically significant. CDTD-Agent also did not differ significantly from Doctor 1 or Doctor 2.

#### 3.2.3 Examination appropriateness

CDTD-Agent scored 4.30 ± 0.70 and was rated significantly higher than Doubao (mean difference 0.30, 95% CI 0.13–0.47; adjusted *p* = 0.001), GPT-4o (0.67, 95% CI 0.50–0.84; adjusted *p* < 0.001), and DeepSeek-V3.2-Exp (0.20, 95% CI 0.03–0.37; adjusted *p* = 0.033). CDTD-Agent also scored higher than Doctor 2 (0.33, 95% CI 0.16–0.50; adjusted *p* < 0.001), whereas the difference from Doctor 1 was not significant.

#### 3.2.4 Western medicine treatment appropriateness

The largest between-source differences were observed in this dimension. CDTD-Agent scored 4.07 ± 0.94, compared with 1.37 ± 0.67 for Doubao, 1.50 ± 0.51 for GPT-4o, and 1.60 ± 0.67 for DeepSeek-V3.2-Exp. The corresponding mean differences were 2.70 (95% CI 2.35–3.05), 2.57 (95% CI 2.22–2.92), and 2.47 points (95% CI 2.12–2.82), respectively; all global BH-adjusted *p* values were < 0.001. CDTD-Agent did not differ significantly from Doctor 1 (mean difference 0.03, 95% CI −0.32 to 0.38; adjusted *p* = 0.852) or Doctor 2 (0.13, 95% CI −0.22 to 0.48; adjusted *p* = 0.494).

#### 3.2.5 TCM treatment appropriateness

CDTD-Agent received the highest mean score (4.33 ± 0.66) and was rated significantly higher than Doubao (mean difference 0.60, 95% CI 0.41–0.79), GPT-4o (1.00, 95% CI 0.81–1.19), and DeepSeek-V3.2-Exp (0.50, 95% CI 0.31–0.69; all adjusted *p* < 0.001). CDTD-Agent also scored higher than Doctor 2 (0.30, 95% CI 0.11–0.49; adjusted *p* = 0.003), whereas the difference from Doctor 1 was not statistically significant (0.13, 95% CI −0.05 to 0.32; adjusted *p* = 0.204).

#### 3.2.6 Health management

No significant differences were observed between CDTD-Agent (4.10 ± 0.55) and Doubao (mean difference −0.10, 95% CI −0.29 to 0.09; adjusted *p* = 0.352), GPT-4o (0.17, 95% CI −0.02 to 0.35; adjusted *p* = 0.104), or DeepSeek-V3.2-Exp (−0.17, 95% CI −0.35 to 0.02; adjusted *p* = 0.104). CDTD-Agent was rated significantly higher than Doctor 1 (0.33, 95% CI 0.15–0.52; adjusted *p* < 0.001) and Doctor 2 (0.47, 95% CI 0.28–0.65; adjusted *p* < 0.001).

#### 3.2.7 Evidence adherence

CDTD-Agent received the highest mean score (4.47 ± 0.68). Ratings were significantly higher than those for Doubao (mean difference 0.47, 95% CI 0.22–0.71; adjusted *p* < 0.001), GPT-4o (0.87, 95% CI 0.62–1.11; adjusted *p* < 0.001), DeepSeek-V3.2-Exp (0.80, 95% CI 0.55–1.05; adjusted *p* < 0.001), Doctor 1 (0.30, 95% CI 0.05–0.55; adjusted *p* = 0.028), and Doctor 2 (0.50, 95% CI 0.25–0.75; adjusted *p* < 0.001).

#### 3.2.8 Personalization

CDTD-Agent scored 4.23 ± 0.77. It received significantly higher ratings than GPT-4o (mean difference 0.63, 95% CI 0.47–0.80; adjusted *p* < 0.001) and Doctor 2 (0.50, 95% CI 0.34–0.67; adjusted *p* < 0.001). The LMM also indicated a modest difference versus Doubao (0.20, 95% CI 0.04–0.37; adjusted *p* = 0.028), whereas no significant differences were detected relative to DeepSeek-V3.2-Exp or Doctor 1. In the non-parametric sensitivity analysis, the CDTD-Agent-versus-Doubao contrast was not statistically significant after global FDR correction. Across all 40 focused contrasts, the mixed-effects and non-parametric analyses yielded concordant significance classifications for 39 comparisons.

### 3.3 *Post hoc* backbone-only ablation analysis

After Holm correction across the eight dimension-specific mixed-effects comparisons, CDTD-Agent received significantly higher ratings than the Qwen backbone-only condition in seven of eight dimensions (Table 6; Figure 5). The largest estimated mean difference was observed for Western Medicine Treatment Appropriateness (1.80, 95% CI 1.40–2.20), followed by Safety (1.23, 95% CI 0.92–1.55) and Evidence Adherence (1.10, 95% CI 0.83–1.37); all Holm-adjusted *p* < 0.001. Significant differences also favored CDTD-Agent for Diagnostic Consistency (0.47, 95% CI 0.23–0.71), Examination Appropriateness (0.57, 95% CI 0.35–0.79), TCM Treatment Appropriateness (0.83, 95% CI 0.57–1.10), and Personalization (0.73, 95% CI 0.49–0.98), all Holm-adjusted *p* < 0.001. Health Management did not differ significantly between CDTD-Agent (4.10 ± 0.55) and the Qwen backbone-only condition (3.93 ± 0.45; mean difference 0.17, 95% CI −0.02 to 0.35; Holm-adjusted *p* = 0.080). Paired Wilcoxon sensitivity analyses produced the same significance classification in all eight dimensions.

**Figure 5:**
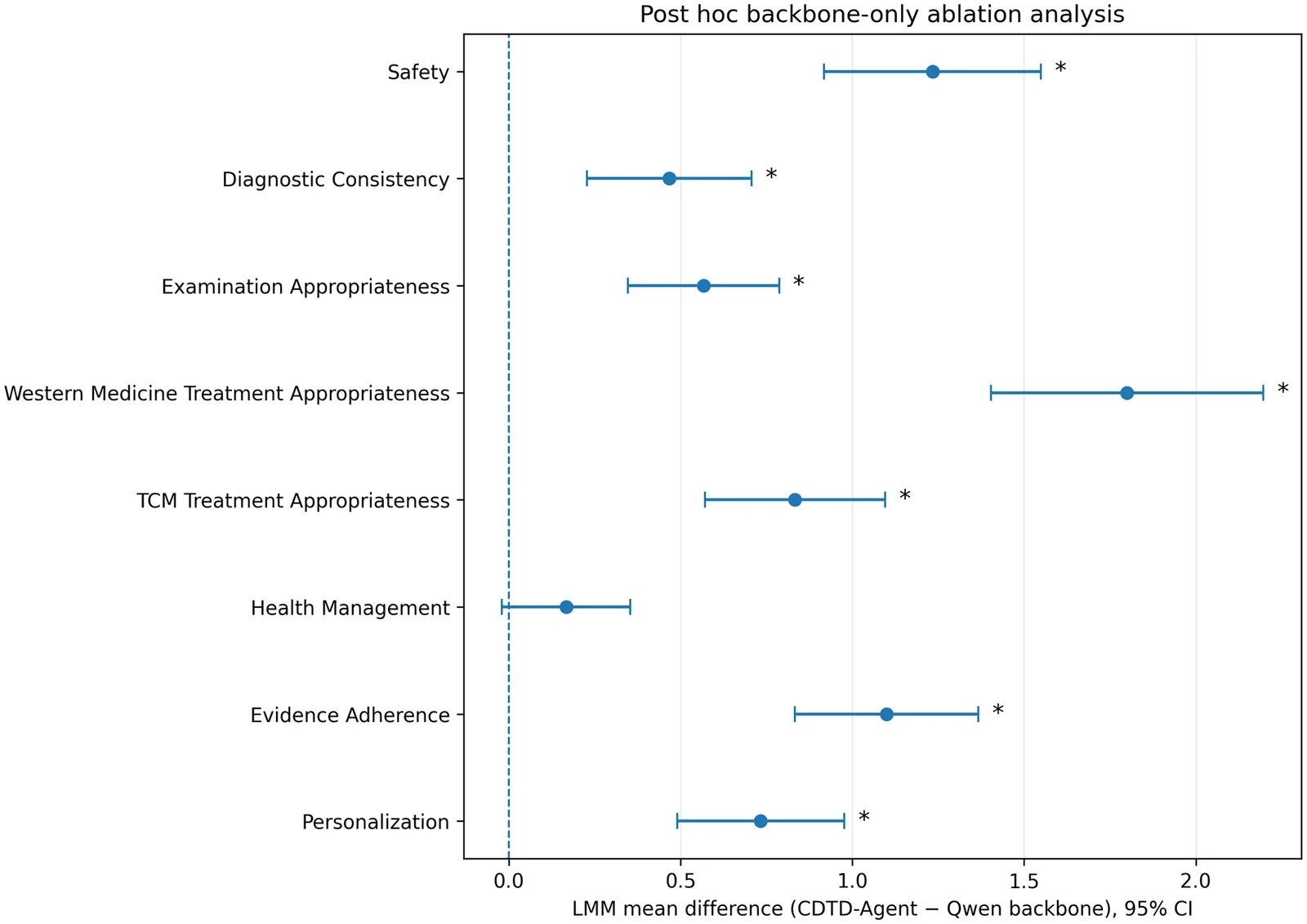
*Post hoc* backbone-only ablation comparing CDTD-Agent with Qwen3-235B-A22B across eight evaluation dimensions. Points represent mixed-effects estimated mean differences (CDTD-Agent minus Qwen backbone-only) with 95% confidence intervals. Asterisks indicate Holm-adjusted *p* < 0.05 across the eight dimension-specific comparisons. Positive values favor CDTD-Agent.

**Table 6:** *Post hoc* backbone-only ablation comparing CDTD-Agent with Qwen3-235B-A22B.

Dimension	CDTD-Agent	Qwen3–235B-A22B backbone-only	Mean difference	95% CI	Holm-adjusted p
Safety	4.33 ± 0.71	3.10 ± 0.99	1.23	0.92 to 1.55	<0.001
Diagnostic Consistency	4.20 ± 0.76	3.73 ± 0.52	0.47	0.23 to 0.71	<0.001
Examination Appropriateness	4.30 ± 0.70	3.73 ± 0.64	0.57	0.35 to 0.79	<0.001
Western Medicine Treatment Appropriateness	4.07 ± 0.94	2.27 ± 0.69	1.80	1.40 to 2.20	<0.001
TCM Treatment Appropriateness	4.33 ± 0.66	3.50 ± 0.68	0.83	0.57 to 1.10	<0.001
Health Management	4.10 ± 0.55	3.93 ± 0.45	0.17	–0.02 to 0.35	0.080
Evidence Adherence	4.47 ± 0.68	3.37 ± 0.49	1.10	0.83 to 1.37	<0.001
Personalization	4.23 ± 0.77	3.50 ± 0.68	0.73	0.49 to 0.98	<0.001

Values are mean ± SD. Mean differences are expressed as CDTD-Agent minus Qwen3–235B-A22B backbone-only; positive values favor CDTD-Agent. Dimension-specific linear mixed-effects models included system condition as a fixed effect and evaluator as a random intercept. The eight *p*-values were adjusted using the Holm procedure. Paired Wilcoxon signed-rank sensitivity analyses with Holm correction yielded the same significance classification for all eight dimensions.

### 3.4 *Post hoc* structured qualitative review of western medicine treatment outputs

The *post hoc* structured review showed that all three general-purpose LLMs provided specific Western medicine management recommendations, but their coverage and adequacy differed across the eight domains (Supplementary Table S1). GPT-4o had no domains coded as adequately addressed; notable omissions involved thyroid abnormality management, diabetes complication screening, hyperuricemia management, and medication safety/follow-up monitoring. DeepSeek-V3.2-Exp likewise had no domains coded as adequately addressed; gaps were observed in dyslipidemia, PCOS, and hyperuricemia management, while several other domains were only partially addressed or included potentially questionable elements. Doubao had five domains coded as adequately addressed, with remaining limitations in glycemic treatment intensity, diabetes complication screening, and hyperuricemia management.

None of the three outputs represented a simple refusal to provide Western medicine management. Doubao had the broadest domain coverage in the qualitative review despite the lowest original Western Medicine Treatment Appropriateness mean score (1.37).

## 4 Discussion

In this blinded single-case pilot evaluation, CDTD-Agent received significantly higher expert ratings than all three general-purpose LLMs in five of eight exploratory dimensions: safety, examination appropriateness, Western medicine treatment appropriateness, TCM treatment appropriateness, and evidence adherence. No general advantage over the three LLMs was observed for health management, while findings for diagnostic consistency and personalization varied by comparator. Because the evaluation involved one standardized T2DM scenario and one fixed output per source, these findings are preliminary and scenario-specific rather than estimates of general model performance.

The observed rating pattern is consistent with the potential value of domain-specific knowledge enhancement in medical-AI systems. General-purpose LLMs show promise in medicine but continue to raise concerns about factual reliability, transparency, and safety (23–25), and comparative clinical-examination studies demonstrate substantial performance variability across models (26). CDTD-Agent was designed to address these weaknesses by retrieving evidence from curated textbooks, clinical guidelines, and expert-reviewed cases before generation. Such evidence-grounded decision-support architecture is consistent with broader calls for careful workflow integration and validation of medical AI systems (27). In the tested case, CDTD-Agent received the highest expert rating for Evidence Adherence. However, evidence access was not condition-matched: retrieved evidence was available to CDTD-Agent as part of the framework, whereas the comparator systems did not receive the same evidence package. The observed difference therefore represents a framework-level difference in expert-rated evidence adherence and should not be interpreted as an isolated causal effect of retrieval. Moreover, Evidence Adherence was an expert-rated overall construct rather than a citation-accuracy or retrieval-quality verification metric.

The role-structured multi-agent design may also have contributed to the observed framework-level differences. Integrated diabetes care requires simultaneous consideration of biomedical diagnosis, pharmacotherapy, TCM syndrome differentiation, and lifestyle intervention (4, 5), and AI-assisted TCM research is increasingly exploring personalized decision support across these knowledge domains (28). In CDTD-Agent, endocrinology, TCM, and health-management roles separated discipline-specific reasoning before synthesis. In the partial ablation, the full framework received significantly higher ratings than backbone-only generation in seven of eight dimensions, whereas Health Management did not differ significantly. This pattern suggests that the added framework may be most relevant to tasks requiring structured clinical reasoning, treatment integration, safety checks, and evidence grounding, while common lifestyle-management advice may be less dependent on these components. Because retrieval, role structuring, and synthesis were removed together, however, their independent contributions cannot be separated by this experiment.

Comparisons with physician-generated recommendations should be interpreted cautiously (29). Several CDTD-Agent-versus-physician contrasts were not statistically significant, including Western Medicine Treatment Appropriateness for both physicians. However, the study was not designed as a non-inferiority or equivalence trial. Absence of a statistically significant difference therefore should not be interpreted as evidence of clinical equivalence to physicians; it indicates only that a difference was not detected for the fixed outputs evaluated in this single scenario.

The *post hoc* qualitative review provides additional context for the low Western Medicine Treatment Appropriateness ratings of the general-purpose LLM outputs. The review indicated heterogeneous content limitations rather than a uniform refusal to provide treatment. GPT-4o and DeepSeek-V3.2-Exp showed multiple areas of incomplete management, whereas Doubao covered more of the predefined management domains but still had gaps in glycemic treatment and complication screening. Importantly, Doubao had the broadest domain coverage in the qualitative review despite the lowest original numerical score. Because evaluator-specific reasons were not collected during the original scoring, the qualitative review describes content characteristics of the fixed outputs but cannot reconstruct why the original 30 evaluators assigned their individual scores.

Taken together, these findings position this work as a pilot evaluation of an integrative medical-AI framework rather than evidence of autonomous clinical capability. At its current stage, CDTD-Agent is best considered a decision-support framework for generating integrated care recommendations, not an autonomous diagnostic or treatment system (30, 31).

This study has several limitations. First, this was a single-case pilot evaluation. The 30 experts provided repeated assessments of fixed outputs for one clinical scenario; they increased the number of expert ratings, not the number of independent clinical cases or model generations. Accordingly, the observed differences apply only to the tested scenario and cannot establish performance variability or generalizability across diabetes phenotypes, comorbidities, complications, TCM presentations, or clinical settings. Second, the endpoint was expert scoring of generated recommendations rather than patient outcomes, clinician behavior, adverse events, or workflow efficiency.

Third, the quality of a retrieval-augmented system depends on the completeness, currency, and indexing quality of its knowledge base (18), and practical medical-AI implementation requires attention to data quality and workflow integration (27). The standardized test case itself was not included in the 183-case retrieval corpus, and no separately entered follow-up record of the same patient was present. However, the overlap assessment was retrospective, a formal algorithmic near-duplicate analysis was not performed, and the exact ranked evidence fragments used during the original generation could not be reconstructed with sufficient certainty. Fourth, model outputs may be affected by prompt templates, model versions, retrieval thresholds, reranking strategies, and decoding settings. The common external evaluation prompt was preserved and matched across AI systems, but low-level decoding parameters were unavailable for the web-based comparator systems, and the exact decoding configuration and verbatim internal role-specific prompt templates used by CDTD-Agent were not retained. Only one output was generated from each AI system, so run-to-run variability was not quantified. Fifth, Evidence Adherence was based on expert judgment and should not be conflated with citation accuracy or retrieval quality. No independent citation-level verification or prospective retrieval-relevance assessment was conducted, and a retrospective retrieval-level audit was not possible because the original ranked evidence fragments could not be reliably reconstructed.

Sixth, the backbone-only ablation was *post hoc* and partial. It compared the complete framework with direct Qwen backbone generation while simultaneously removing retrieval, role-structured reasoning, and multi-role synthesis, and the backbone-only output was rated in a follow-up evaluation rather than concurrently with the original six outputs. Seventh, single-evaluator absolute agreement varied substantially across dimensions, with particularly low ICC(A,1) for diagnostic consistency, although reliability improved when ratings were aggregated across the 30-evaluator panel. Eighth, the *post hoc* qualitative review was conducted by two clinician reviewers after the quantitative evaluation, rather than by the original 30 evaluators, using a coding framework developed for retrospective content characterization. Finally, expert ratings do not establish prospective safety, clinical effectiveness, implementation feasibility, or real-world utility.

Future work should evaluate CDTD-Agent using larger and more diverse case sets across multiple institutions. External validation should include heterogeneous real-world electronic health record–derived cases and clinically challenging scenarios, including incomplete information, conflicting symptoms, complex comorbidities, and high-risk medication decisions (32). When appropriate, future evaluation should also incorporate multimodal clinical inputs (33). Prospective studies are needed to determine whether CDTD-Agent improves clinician decision quality, reduces workload, or improves patient outcomes without increasing safety risk. Future studies should extend the partial backbone-only analysis using prospective factorial ablation designs, including retrieval versus no retrieval and multi-agent versus single-agent generation, with repeated generations under controlled sampling settings to isolate individual component contributions. Future retrieval evaluations should prospectively archive query-level retrieval logs and independently assess retrieval relevance, source correctness, and claim–evidence correspondence. Finally, human-in-the-loop interaction, knowledge-base updating, governance, accountability, and transparency mechanisms should be developed before clinical deployment (34).

## 5 Conclusion

CDTD-Agent is a retrieval-augmented multi-agent framework for integrated diabetes care recommendations. In this blinded single-case pilot evaluation, its fixed output received significantly higher expert ratings than all three general-purpose LLM outputs in five of eight exploratory dimensions, while the *post hoc* backbone-only ablation favored the full framework in seven of eight dimensions. These scenario-specific findings support further evaluation of the complete framework but do not establish generalizable clinical performance, superiority or equivalence to physicians, or the independent effects of retrieval and multi-agent components. Multicase, repeated-generation, factorial-ablation, external, and prospective clinical validation is needed before deployment.

## Data Availability

The common external evaluation prompt and final consensus coding from the post hoc qualitative review are provided in the Supplementary Material. The de-identified standardized test case, fixed recommendation outputs used for expert evaluation, evaluator-level ratings from the primary six-source experiment, evaluator-level ratings from the post hoc Qwen3-235B-A22B backbone-only ablation, original qualitative-review records, and statistical analysis materials are available from the corresponding authors upon reasonable request, subject to applicable institutional data-governance and data-protection requirements. The curated 183-case retrieval corpus contains clinical case materials and is subject to privacy, data-governance, and source-redistribution restrictions; therefore, the full corpus is not publicly released. Its composition and source categories are described in the Methods and Table 1. The exact ranked retrieval logs and evidence fragments used during the original CDTD-Agent generation were not retained with sufficient provenance and are therefore unavailable for retrospective release. Although the common external evaluation prompt was preserved, the verbatim internal role-specific prompt templates and exact decoding parameters used during the original CDTD-Agent generation were not retained and could not be reliably reconstructed retrospectively.
