# Supplementary material for: CDTD-Agent: a retrieval-augmented multi-agent digital health framework for integrated diabetes care: a single-case expert-rated pilot study
Source: Front Digit Health. 2026 Sep 16;8:1923975. doi: 10.3389/fdgth.2026.1923975 (PMC13623845; doi:10.3389/fdgth.2026.1923975)
Supplement: Supplementary file 1 [file Datasheet1.docx]

# **Supplementary Appendix S1. Common External Evaluation Prompt**

The following Chinese-language prompt was used as the common external evaluation prompt for all AI systems in the primary comparison and for the Qwen3-235B-A22B backbone-only ablation.

请根据如下病历信息，制定一份全面、合理的诊疗方案。

要求涵盖以下三个方面：

1. 西医诊疗：包括进一步评估、必要检查、诊断判断及治疗建议；
2. 中医诊疗：包括中医辨证、治法及方药或其他中医干预建议；
3. 健康管理：包括饮食、运动、睡眠、心理、危险因素控制及随访管理等建议；
4. 各部分应结合患者具体情况，避免泛泛而谈，并说明重要治疗决策的主要依据；
5. 如患者存在需要进一步确认的信息、治疗禁忌或潜在风险，请明确指出。

**English translation:**

Please formulate a comprehensive and appropriate diagnosis and treatment plan based on the following medical record information. The response should cover the following aspects: (1) Western medicine care, including further evaluation, necessary examinations, diagnostic assessment, and treatment recommendations; (2) traditional Chinese medicine care, including TCM syndrome differentiation, therapeutic principles, and recommendations regarding formulas, herbal medicines, or other TCM interventions; (3) health management, including recommendations regarding diet, exercise, sleep, psychological well-being, risk-factor control, and follow-up management; (4) each component should be tailored to the patient's specific clinical situation rather than providing generic advice, and the main rationale for important treatment decisions should be stated; and (5) if additional information needs to be confirmed, or if treatment contraindications or potential risks exist, these should be explicitly identified.

# **Supplementary Table S1. Final Consensus Coding From the Post Hoc Structured Qualitative Review of Western Medicine Treatment Outputs**

| **Management domain** | **GPT-4o** | **DeepSeek-V3.2-Exp** | **Doubao** |
| --- | --- | --- | --- |
| Glycemic treatment intensity | P/Q — Metformin was proposed, but treatment intensification was limited relative to the marked hyperglycemia, and individualized medication-safety considerations in the setting of abnormal liver tests were insufficient. | P — Hyperglycemia was recognized and GLP-1–based therapy/SGLT2 inhibition was suggested, but a concrete treatment regimen and escalation strategy were not provided. | P/Q — A specific glucose-lowering regimen was provided, but treatment intensification and individualized medication-safety considerations remained incomplete. |
| Thyroid abnormality management | N — The marked TSH abnormality was not addressed. | P — The marked TSH abnormality was recognized and further differentiation was suggested, but no clear repeat testing, additional work-up, or treatment pathway was provided. | A — The thyroid abnormality was recognized and a treatment plan was provided. |
| Hypertriglyceridemia/dyslipidemia management | P — Dyslipidemia was recognized and lipid control was mentioned, but no specific treatment and monitoring plan was provided for the marked hypertriglyceridemia. | N — Hyperlipidemia was recognized diagnostically, but no treatment plan was provided. | A — Dyslipidemia was recognized and a specific treatment and monitoring plan was provided. |
| Fatty liver/liver-function management | P/Q — Fatty liver was recognized and drug therapy was suggested, but management was relatively narrow and did not sufficiently integrate weight reduction, metabolic control, and fibrosis risk. | P — Fatty liver and abnormal liver tests were recognized and further assessment/follow-up was suggested, but the overall management pathway remained incomplete. | A — Fatty liver and abnormal liver tests were recognized and an overall treatment and follow-up plan was provided. |
| PCOS management | P — PCOS was recognized and oral contraceptive treatment was suggested, but individualized indications, safety, and related risks were insufficiently assessed. | N — PCOS was recognized and follow-up was suggested, but no specific treatment plan was provided. | A — PCOS was recognized and a specific management plan was provided. |
| Diabetes complication screening | N — Standard baseline complication screening was not adequately addressed. | P/Q — Detailed complication-screening suggestions were provided, but some proposed vascular screening was considered potentially broader than necessary for this presentation. | N — Standard baseline complication screening was not adequately addressed. |
| Hyperuricemia management | N — Hyperuricemia was not specifically managed. | N — Hyperuricemia was recognized diagnostically, but no management plan was provided. | P — Hyperuricemia was recognized and basic management was proposed, but the plan remained incomplete. |
| Medication safety and follow-up monitoring | N — Medication interactions, contraindications, and individualized safety monitoring were not adequately addressed. | P/Q — Some medication-safety and monitoring issues were addressed, but coverage was incomplete and several recommendations remained open to clinical scrutiny. | A — Medication safety and follow-up monitoring were addressed in a relatively complete manner. |

Note. A, adequately addressed; P, partially addressed; N, not addressed; Q, potentially questionable recommendation; P/Q, both partially addressed and potentially questionable. The coding was developed for this post hoc descriptive review and is intended to contextualize, not causally explain, the original expert ratings.

# **Supplementary Table S2. Inter-Rater Reliability by Evaluation Dimension**

| **Dimension** | **ICC(A,1)** | **95% CI** | **ICC(A,30)** | **95% CI** |
| --- | --- | --- | --- | --- |
| Safety | 0.411 | 0.195–0.813 | 0.954 | 0.879–0.992 |
| Diagnostic Consistency | 0.045 | 0.009–0.259 | 0.585 | 0.211–0.913 |
| Examination Appropriateness | 0.077 | 0.025–0.349 | 0.713 | 0.430–0.942 |
| Western Medicine Treatment Appropriateness | 0.771 | 0.556–0.953 | 0.990 | 0.974–0.998 |
| TCM Treatment Appropriateness | 0.126 | 0.044–0.478 | 0.812 | 0.579–0.965 |
| Health Management | 0.074 | 0.024–0.345 | 0.707 | 0.416–0.941 |
| Evidence Adherence | 0.111 | 0.037–0.450 | 0.789 | 0.530–0.961 |
| Personalization | 0.070 | 0.022–0.327 | 0.693 | 0.404–0.936 |

Note. ICC values were calculated using a two-way absolute-agreement model. ICC(A,1) represents single-evaluator absolute agreement, and ICC(A,30) represents reliability of the mean rating aggregated across 30 evaluators. CI, confidence interval.
